# A Novel Stool Methylation Test for the Non-Invasive Screening of Gastric and Colorectal Cancer

**DOI:** 10.3389/fonc.2022.860701

**Published:** 2022-03-28

**Authors:** Liang Ma, Jian Gong, Meimei Zhao, Xiaomu Kong, Peng Gao, Yongwei Jiang, Yi Liu, Xiaoyan Feng, Shuang Si, Yongtong Cao

**Affiliations:** ^1^ Department of Clinical Laboratory, China-Japan Friendship Hospital, Beijing, China; ^2^ Department of Research and Development, Apexbio Biotechnology (Suzhou) Co., Ltd., Suzhou, China; ^3^ Department of General Surgery, China-Japan Friendship Hospital, Beijing, China

**Keywords:** multi-gene, gastric and colorectal cancer, methylation, early detection, support vector machine

## Abstract

**Background:**

Because of poor compliance or low sensitivity, existing diagnostic approaches are unable to provide an efficient diagnosis of patients with gastric and colorectal cancer. Here, we developed the ColoCaller test, which simultaneously detects the methylation status of the SDC2, TFPI2, WIF1, and NDRG4 genes in stool DNA, to optimize the screening of gastric and colorectal cancer in high-risk populations.

**Methods:**

A total of 217 stool samples from patients with gastrointestinal cancer and from patients with negative endoscopy were prospectively collected, complete with preoperative and postoperative clinical data from patients. The methylation of these samples was detected using ColoCaller, which was designed by selecting CpGs with a two-step screening strategy, and was interpreted using a prediction model built using libSVM to evaluate its clinical value for gastric and colorectal cancer screening.

**Results:**

Compared to pathological diagnosis, the sensitivity and specificity of the ColoCaller test in 217 stool DNA samples were 95.56% and 91.86%, respectively, for colorectal cancer, and 67.5% and 97.81%, respectively, for gastric cancer. The detection limit was as low as 1% in 8 ng of DNA.

**Conclusion:**

In this study, we developed and established a new test, ColoCaller, which can be used as a screening tool or as an auxiliary diagnostic approach in high-risk populations with gastric and colorectal cancer to promote timely diagnosis and treatment.

## Introduction

Gastric and colorectal cancer (GCC) are important systemic diseases that affect human health. According to statistics, the number of new cases and deaths from GCC was 3,020,693 and 1,703,966, which represented 15.6% and 17.1% of total cancer in the world, respectively, in 2020 (1). Globally, about 48.6% of gastric cancer (GC) and 30.6% of Colorectal cancer (CRC) deaths occur in China (2). Most CRC patients are diagnosed with advanced cancer, of whom the 5-year survival rate is only 14%. The 5-year survival can be as high as 90% if the disease is detected at stage I, suggesting a better survival outcome is associated with early detection (3, 4). Likewise, the 5-year survival rate of patients with GC is up to 95% when patients are diagnosed as pT1a non-metastatic carcinomas, but below 1% for N3 cases (5). Therefore, it is of great significance to early detect GCC to decrease the morbidity and improve patients’ survival.

In recent years, endoscopy inspection is the most accurate method for early screening of GCC, but it is an invasive examination, with a certain risk of bleeding and perforation, poor patient compliance, and is not suitable for large-scale screening. Although there are currently many serum biomarkers used for screening early cancer, they do not meet the high sensitivity and specificity required for detecting GCC. Therefore, it is urgent to establish a non-invasive, convenient, and accurate screening method for the prevention and treatment of gastrointestinal cancer. Both of CRC and GC progresses through a multistep process that involves accumulation of both genetic and epigenetic alterations (6). As an early event in tumorigenesis and development, abnormal DNA methylation has been found to be strongly related to the occurrence of cancer and has great potential to become a tumor diagnostic biomarker (7–10). Although many sources, including serum/plasma and gastric washing lavage, could be used to access methylation analysis, the stool is perhaps the most convenient and promising source for cancer screening, especially for CRC screening. There are 10^9^ epithelial cells that are shed from the normal mucosa every day. Due to the rapid renewal rate of tumor epithelial cells, at least 1% of cells fall into the intestine every day and are excreted from the feces (11, 12). In fact, many studies have shown that methylation in stool DNA is suitable for early detection of CRC and has a higher sensitivity and specificity than fecal occult blood test (FOBT) and colonoscopy (13–15). Ahlquist et al. reported that the sensitivity of DNA methylation of 4 genes (NDRG4, BMP3, TFPI2, vimentin) for patients with CRC and in patients with advanced adenomas (AA) (>1 cm) patients was 85% and 54%, respectively, and the specificity was 90% (16). Currently, several methylation tests have already been approved by the FDA or NMPA for the screening of CRC (17–19). The SEPT9 methylation kit was first approved based on epigenetic changes in blood for CRC screening. A meta-analysis showed that SEPT9 methylation has a relatively poor sensitivity of 73-78% and 8-31% in CRC and AA screening respectively (19). Therefore, the value of SEPT9 methylation for early CRC screening is questionable (20). With further research, additional methylated genes, were found to be useful in CRC screening, such as SFRP2, SDC2, and WIF1. However, early detection of GC by DNA methylation still has many challenges to overcome. There are few reports and products on the detection of GC using stool DNA methylation.

In this study, we explored the feasibility of DNA methylation for detection of GCC, and established a new assay, the ColoCaller test, which combined the detection of SDC2, TFPI2, WIF1, and NDRG4 methylation, and evaluated its clinical performance for early detection of GCC.

## Material and Methods

### Sample Collection and Processing

**Figure 1:**
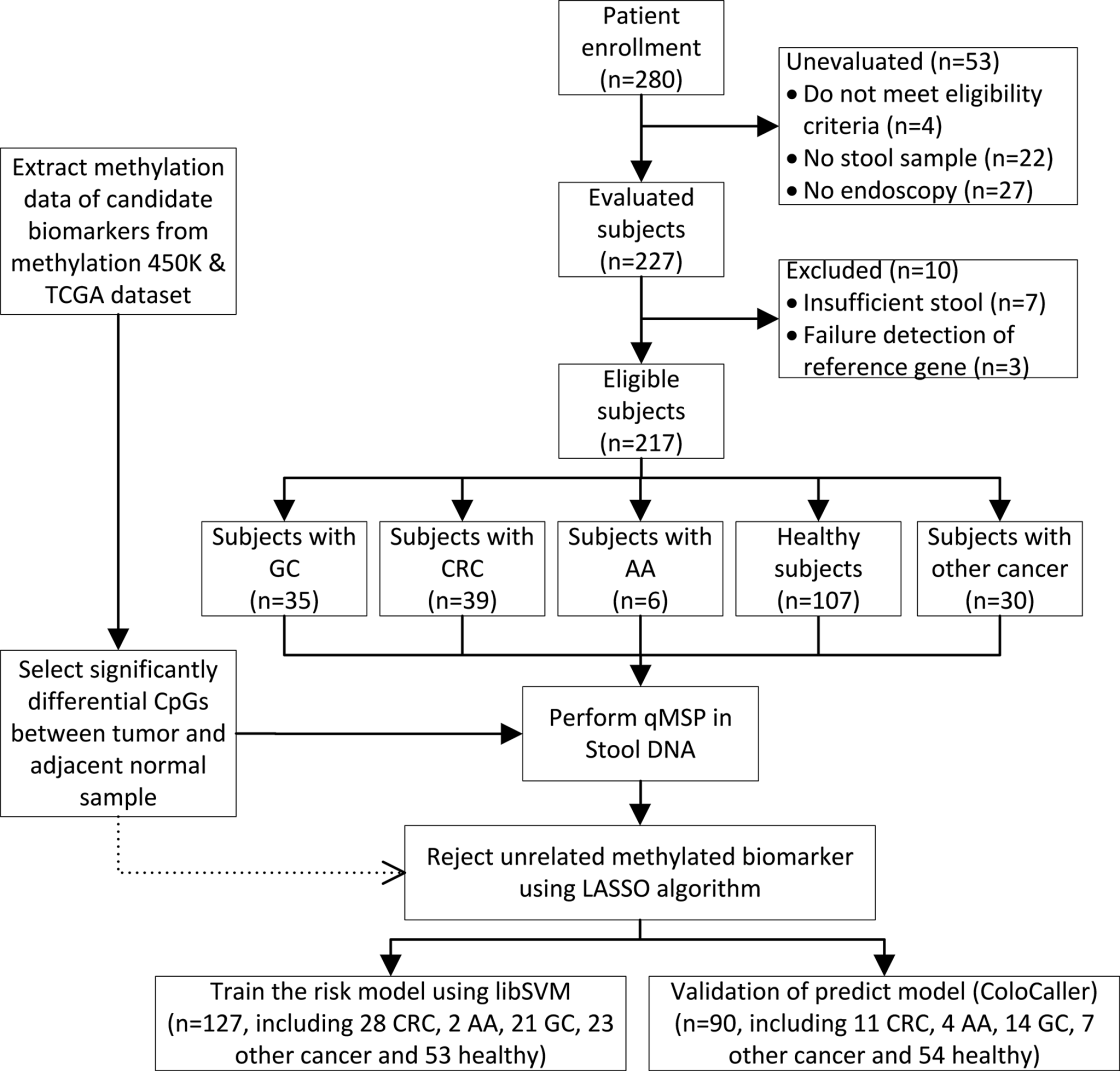
Study flowchart. GC, gastric cancer; CRC, colorectal cancer; AA, advanced adenomas; LASSO, Least Absolute Shrinkage and Selection Operation.

A total of 280 participants who were scheduled to undergo an endoscopy examination or with confirmed digestive tract cancer including CRC, advanced adenoma (AA), GC, other digestive tract cancer, and healthy individuals, were enrolled at the China-Japan Friendship Hospital and were asked to collect stool samples at home from July 2019 to November 2020 (**Figure 1**). Sixty-three patients with incomplete pathological data, insufficient samples, or that withdrew from the study were excluded and 217 patients with confirmed colonoscopy and/or pathological diagnosis were enrolled in this study. There was no history of infection or second malignancies in the patients and none received any preoperative radiotherapy, chemotherapy, or endocrine therapy. All patients provided their informed consent in writing. This study was approved by the Ethics Board of the Institute of China-Japan Friendship Hospital and was carried out in accordance with the specifications of the Declaration of Helsinki. Each patient was asked to collect a 3 g stool samples in a Stool Collection Tube (Apexbio, Suzhou, China). Samples were delivered to the laboratory at room temperature or stored at -20°C until further analysis. The CRC cell line HCT116 was purchased from the Peking Union Cell Resource Center (Beijing, China) and was cultured in Dulbecco’s modified Eagle’s medium (DMEM) at 37°C in 5% CO_2_.

### DNA Extraction and Bisulfite Conversion

All DNA was extracted using a stool DNA extraction kit (Apexbio, Suzhou, China) according to the manufacturer’s instructions. After measuring the concentration of DNA using the Qubit dsDNA HS Assay (Invitrogen, Carlsbad, CA, US), 800 ng of DNA was treated with sodium bisulfite using the DNA methylation kit (Apexbio, Suzhou, China) following the manufacturer’s instructions. The converted DNA was used immediately or stored at -20°C until use.

### Selection of the Candidate Methylation Region

To comprehensively explore and verify the abnormal gene methylation in CRC, publicly available data including The Cancer Genome Atlas (TCGA) datasets (TCGA-COAD.methylation450; platform: Illumina Infinium Human Methylation 450K BeadChip, including 462 primary tumors and 85 normal tissues) and GEO datasets (GSE101764, GSE139404, GSE68060, GSE149282, and GSE129364; platform: Illumina Infinium Human Methylation 450K BeadChip, including 149/112 tumor-adjacent/tumor samples, 20/40 normal/tumor samples, 36/82 normal/tumor samples, 12/12 normal/tumor samples, and 3/69 normal/tumor samples, respectively) were downloaded from TCGA portal (https://portal.gdc.cancer.gov) and the Gene Expression Omnibus (GEO) database of the National Center for Biotechnology Information (http://www.ncbi.nlm.nih.gov/geo), respectively. After excluding unqualified samples (only Stage I/II samples were selected), there were 268 primary carcinoma samples and 239 normal samples. After normalization using ‘champ.norm’ function in the package R ‘Champ’ and filtration by the probe annotated by the corresponding annotation files for each data set, intact data of candidate methylated genes (SDC2, NDRG4, KCNQ5, WIF1, TFPI2, and ALX4), which were selected based on a PubMed library literature search, were collected and analyzed using the package R ‘Champ’ with an adjusted p-value <0.05 and a deltaBeta value >0.2 (13, 21–23).

### Establishment and Verification of the ColoCaller Test

The primers and probes for the methylated genes (SDC2, WIF1, TFPI2, NDRG4, KCNQ5 and ALX4) were designed with MethPrimer software (24). All oligonucleotides in this study were synthesized by Sangon Biotech Co., Ltd (Sangon, Shanghai, China). The triple quantitative methylation specific PCR (qMSP) reaction of ColoCaller test (Apexbio, Suzhou, China) was performed in a 20 μL reaction containing 1X PerFecTa Multiplex qPCR ToughMix (Quanta Biosciences, Gaithersburg, USA), 400 nM of each forward primer and reverse primer, 200 nM of each probe, and 10 μL the converted DNA. This was programmed as: 95°C for 3 min, 5 cycles of 94°C for 10 s, 58°C for 30 s, followed by 45 cycles of 94°C for 10 s, 55°C for 30 s on the Cielo DX3 PCR System (Apexbio, Suzhou, China) and the 7500 Real-time System (ThermoFisher, US) according to the manufacturers’ instructions. All experiments were replicated to ensure reproducibility, and were performed under blind control.

Least Absolute Shrinkage and Selection Operation (LASSO) regression analysis can screen multiple eigenvalues and retain key eigenvalues. We used LASSO regression analysis to analyze 6 methylated genes in this study using the R package ‘glmnet’ (25). The Ct value of qMSP in 217 stool DNA was collected and randomly assigned to the training set (70%) and the validation set (30%) to screen the optimal methylated genes using the R package ‘glmnet’ with 10-fold cross-validation. The Ct value of each gene was transformed into the ‘0/1’ format according to its cutoff value, which was obtained by ROC analysis in advance. If the Ct value was greater than the corresponding cutoff value or there is no amplification curve, we transformed the Ct value to 0 and input it into subsequent analysis. Otherwise, we transformed the Ct value to 1. Sensitivity, specificity, and AUC were evaluated using the ‘pROC’ R package.

Subsequently, nonsignificant methylated genes were rejected and the amplification value of specific methylation genes was used to build the prediction model based on the support vector machine (SVM) polynomial classifier and kernel algorithm using the package R ‘e1071’ to identify the risk of early CRC (26, 27). In this study, the amplification data were randomly divided into the training group (n=127) and the validation group (n=90). The logistic regression formula is as follows:


(1) $$\text{Score}=W^{\text{T}}\text{X}+\text{b}$$

Where the normal vector w= (w_1_; w_2_; …; w_d_), X= (X_1_; X_2_; …; X_n_) was described by n genes, and X_i_ was the value of X in the i-th attribute, which was transformed from the value of Ct in the test (27).


(2) $$\text{P}={\text{e}}^{\text{Score}}\text{/(1}+{\text{e}}^{\text{Score}})$$

where P was the risk index for CRC. The threshold of P value was assessed by ROC analysis for optimal sensitivity and specificity discriminating GCC patients from healthy participants. Each sample was considered ‘negative’ with high risk of GCC if the P value was < 0.087, otherwise a ‘positive’ value for high risk of GCC was scored.

### ColoCaller Test Performance Analysis

To determine the limit of detection (LoD) of the ColoCaller test, different amounts (10 ng, 8 ng, and 6 ng) of fully-methylated genomic DNA (CRC cell line HCT116 DNA) and unmethylated stool DNA from healthy individuals was mixed in a mixture of 5%, 1%, and 0.5%, respectively. The methylation status of SDC2, WIF1, TFPI2, and NDRG4 genes was confirmed by Sanger sequencing. A total of 800 ng of mixed DNA was bisulfite converted and 10 replicates of each concentration series were amplified using two qPCR platforms with 3 batches of reagents to determine LoD, which was estimated by Probit analysis. Precision analysis was carried out using 10%, 5%, and 0% methylated DNA prepared by mixing DNA from HCT116 and DNA and healthy individuals DNA with 20 replicates using two qPCR platforms, respectively. The positive/negative accuracy rate was performed in methylated/non-methylated DNA mixed with different interruption materials (animal/plant DNA, microbic DNA, drugs, DNA with other methylated genes).

### Statistical Analyses

All statistical analyses were performed using R version 3.6.1 and SPSS software (SPSS 22.0, IBM, USA). The Ct values of the corresponding methylated genes were input to construct the prediction model to evaluate the risk of CRC. The Ct value can be calculated only when the amplification curve has a significant exponential growth period or shows an S-type amplification. Sensitivity, specificity, and area under the curve (AUC) were used to evaluate the performance of the prediction models. All statistical tests were two-sided and a P-value of <0.05 was considered statistically significant.

## Results

### Clinical Characteristics

In this study, a total of 217 participants with confirmed colonoscopy and/or pathological diagnosis (including 39 patients with CRC, 6 patients with AA, 35 patients with GC, 30 patients with other digestive tract cancers, and 107 healthy controls) were enrolled. The clinicopathological characteristics of the participants are shown in **Table 1**.

**Table 1:** Clinicopathological characteristic of patients analyzed.

Variable	No. of patients (n, %)	No. of ColoCaller test positive (n, %)
**Total**	217	57 (26.27)
**Colorectal cancer**	39 (17.97)	37 (94.87)
Sex		
Male	28 (71.79)	28 (100)
Female	11 (28.21)	9 (81.82)
Age		
40-50	31 (79.49)	29 (93.55)
>50	8 (20.51)	8 (100)
Location		
Left	35 (89.74)	33 (94.29)
Right	4 (10.26)	4 (100)
TNM stage		
I	9 (23.07)	8 (88.89)
II	16 (41.03)	15 (93.75)
III/IV	14 (35.90)	14 (100)
**Adenomas (>1 cm)**	6 (2.76)	6 (100)
**Gastric cancer**	35 (16.13)	11 (31.43)
**Pancreatic cancer**	12 (5.53)	0 (0)
**Liver cancer**	6 (2.76)	0 (0)
**Esophagus cancer**	6 (2.76)	0 (0)
**Gallbladder cancer**	6 (2.76)	1 (16.67)

### Analytical Performance of the ColoCaller Test

Methylated genes were detected (10/10) with 10 replicates using two different qPCR platforms with an 8 ng sample of 1% methylated DNA. Therefore, the LoD was set at 1% for an 8-ng sample. The precision of the ColoCaller test was determined using a low positive control (8 ng, 5%) and a median positive control (8 ng, 10%), according to the CLSI guidelines EP15-A3. We replicated the test 20 times on two qPCR platforms and the CV of the detected Ct was less than 5.0%, respectively. The result of interference experiment showed that none of the drugs had any effect to the detection, except for 9.23 mg/mL berberine.

### Establishment of the ColoCaller Test

In this study, we used a two-step screening strategy to select methylation biomarkers and then used machine learning methods to build a predictive model. In summary, we first extracted the methylation data of specific genes from public databases, and after analysis and sorting, we selected CpGs that were significantly different in tumors and normal adjacent tissues. To select the CpGs of candidate methylated genes filtered with the annotation file and normalized to eliminate the cross-batch effect, differential analysis was performed on the tumor and adjacent normal tissue. A total of 197 CpGs were obtained, including 20 significantly downregulated CpGs, 69 stable CpGs, and 108 significantly upregulated CpGs (deltaBeta>0.2, adj.P-value<0.05) (**Supplementary Table 1**). In addition, the volcano and heatmap showed differences between the different samples for the same CpGs, which basically confirmed the above results (**Supplementary Figures 1**, **2**). No significant differences in the CpG of the CCND2, NEUROG1, SEPT9, SFRP1, and SFRP2 genes were found after normalization. There were many significantly upregulated CpGs for EYA4 and BMP3, but the consistency between different datasets was very poor. However, the CpG profiles for KCNQ5, WIF1, TFPI2, NDRG4, ALX4, and SDC2 genes were relatively consistent between each dataset (**Supplementary Table 2**). Therefore, the CpGs of KCNQ5, WIF1, TFPI2, NDRG4, ALX4, and SDC2 genes were used to design probes for multitarget detection and to perform the qMSP tests in subsequent studies. Compared to clinical pathological data, ALX4 and KCNQ5 CpGs were excluded using the LASSO algorithm, which were of little relevance within the prediction model (**Supplementary Table 3** and **Supplementary Figure 4**). While simplifying the complexity of the model, the selection of markers was further optimized.

**Figure 2:**
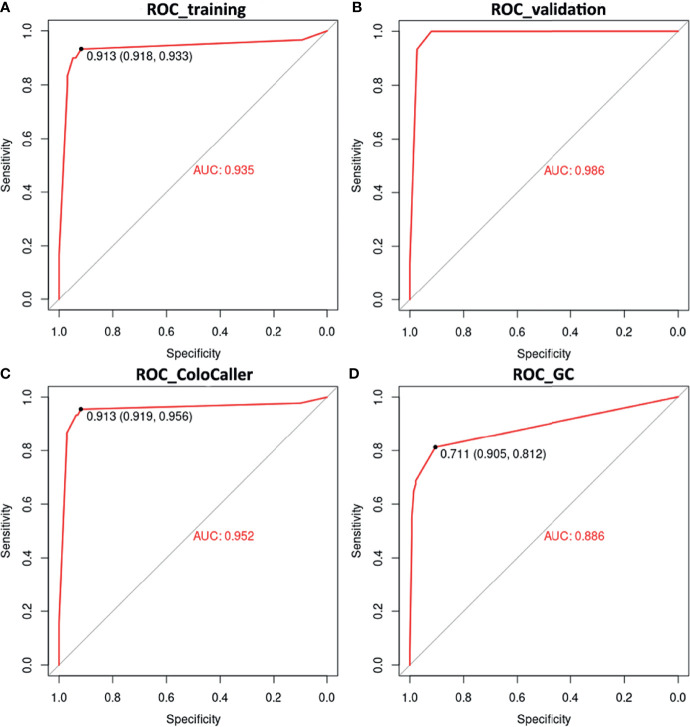
Diagnostic performance of ColoCaller test in 217 stool DNA. Receiver operating characteristics (ROC) curves of ColoCaller test for CRC in the training **(A)**, validation **(B)** and entire **(C)** group. **(D)** Performance of ColoCaller for gastric cancer (GC) detection.

Next, we use SVM to construct a prediction model for the early diagnosis of GCC. The methylation status of SDC2, WIF1, TFPI2, and NDRG4 was randomly divided into a training set (n=127) and a validation set (n=90). Among the two data sets, data from patients with other cancers, especially with digestive tract cancers, were included to further verify the specificity of the ColoCaller test for CRC. The prediction results of the training set showed that the model prediction was good, with the accuracy, sensitivity, and specificity of 92.10% (95%CI 86.10%-96.70%), 93.33% (95%CI 78.67%-98.15%), and 91.75% (95% CI 84.56%-95.76%), respectively, and the AUC area reached 0.94 (**Figure 2** and **Supplementary Table 3**). Consistent with the training set, the AUC of the validation set was 0.99. For the entire cohort of 217 participants, the Kappa value, sensitivity, and specificity of the ColoCaller test for the detection of CRC were 0.80, 95.56% (43/45, 95%CI 85.17%-98.77%), and 91.86% (158/172, 95%CI 86.80%-95.09%), respectively (**Figure 2C** and **Table 2**. For 6 patients with AA, the positive detection rate of methylation was 100%. Furthermore, excluding the patients with other digestive tract cancers, the sensitivity and specificity of the ColoCaller test for CRC screening reached 95.56% (43/45, 95%CI 85.17%-98.77%) and 98.13% (105/107, 95%CI 93.44%-99.49%), respectively (**Supplementary Table 3**).

**Table 2:** Comparison performance of ColoCaller test in CRC.

		Pathological characteristics of CRC		
		+^a^	- ^b^	Total
**ColoCaller test**	**+**	43	14	57
	**-**	2	158	160
	**Total**	45	172	217
**Sensitivity**		95.56% (85.17%-98.77%)		
**Specificity**		91.86% (86.80%-95.09%)		

a , “+” means methylation positive, and b, means methylation negative.

### Methylated SDC2 Gene Alone Was Less Sensitive Than ColoCaller for Early CRC Screening

In this study, we also compared the performance of a single methylated gene with the ColoCaller test. The sensitivity/specificity of SDC2, TIFP2, NDRG4, and WIF1 gene alone in the training set were 90.00%/91.80%, 80.00%/83.50%, 76.70%/95.90%, and 80.00%/92.80%, respectively (**Supplementary Figure 3**). For the SDC2 gene alone, the performance was less efficient than the ColoCaller test, even in the entire cohort with less sensitivity (93.30% vs 95.60%) for CRC screening (**Supplementary Table 3**).

### The ColoCaller Test Could Be Used as an Auxiliary Screening Tool for GCC

Among digestive tract cancers, the methylation profile of GC may be similar to that of CRC. In this study, using the ColoCaller algorithm, 54 patients were detected as having a positive result for GCC methylation (including CRC and GC), with a sensitivity and specificity of 68.75% (54/80) and 97.81% (134/137) (**Table 3** and **Supplementary Table 4**). Furthermore, we also reconstructed the prediction model for GCC, and the result was the same as before, with a specificity of 97.81% (134/137, 95%CI 93.76%-99.25%). It indicated that the ColoCaller test could be used as an auxiliary tool for the screening of GCC.

**Table 3:** Comparison performance of ColoCaller test in GCC.

		Pathological characteristics of GCC			
		+^a^		-^b^	Total
**ColoCaller test**	**+**	54		3	58
	**-**	26		134	159
	**Total**	80		137	217
**Sensitivity**			67.5% (56.64%-77.85%)		
**Specificity**			97.81% (93.76-99.25%)		

a , “+” means methylation positive, and b, means methylation negative.

## Discussion

Endoscopy inspection is currently recognized as the “gold standard” for the screening of gastrointestinal cancer (28). However, as an invasive examination, it will bring certain pain and a higher risk of complications. In addition, medical resources for endoscopy examinations are scarce and unevenly distributed, which cannot yet satisfy population screening. The sixth census showed that there are about 40 million people will require a colonoscopy in China. However, the annual colonoscopy capacity is only 5.38 million and there are less than 40,000 endoscopists (29–31). Currently, a series of studies have found that many genes are hypermethylated in CRC, and some have been used in clinical practice (32, 33). DNA methylation of the stool has already been considered as an effective tool to detect CRC. However, there are few reports on the detection of GC by DNA methylation.

In this study, we investigated the methylation profile in public datasets to identify potential biomarkers for GC and CRC, and the development of a new diagnostic test, ColoCaller. Subsequently, the clinical diagnostic value of ColoCaller was evaluated in 217 stool samples from patients with gastrointestinal cancer and those with negative endoscopy, which were prospectively collected. Several hypermethylated genes (WIF1, SDC2, TFPI2, NDRG4) were screened and compared in tumors and normal tissue of patients with GC and CRC patients, which had great potential to become a diagnostic biomarker. Due to the high acidity in the stomach, previous reports have indicated that DNA may be denatured and cannot be detected in the stool for GC (34). However, the sensitivity of ColoCaller test for GC was 67.5% and the specificity was about 97.81%. These results confirmed that stool DNA obtained from exfoliated cells could be detected from patients with GC, and the ColoCaller test could be used as diagnostic biomarker for GC.

In addition, several reports have been published on the relationship between methylated genes (WIF1, SDC2, TFPI2, NDRG4) and CRC. Some genes have high specificity and low sensitivity for methylation, while others have the opposite. However, the influence of these genes on GC is still unclear. Only methylation of the SDC2 and TFPI2 genes has been reported to be related to GC (35–37). In view of the excellent performance of SDC2 gene methylation in the detection of CRC in previous reports, we analyzed the screening capability of methylated SDC2 alone for CRC. The results showed that the performance was less efficient than that of the ColoCaller test, even in the entire cohort with less sensitivity (93.30% vs 95.60%) for the detection of CRC. Furthermore, we found that a high positive rate of these methylated genes was observed not only in GCC, but also in other gastrointestinal cancers, which indicates that single gene methylation cannot easily distinguish the location of gastrointestinal cancer. Because the DNA of tumor cells that shed stool cannot contain a single gene, the sensitivity and specificity of single gene methylation has not reached a very satisfactory level. Combined multitarget detection has become a common method to improve detection sensitivity (25, 38–41). A previous study reported that the sensitivity of the stool DNA methylation test using single and multiple genes was 48% and 77.8%, and the specificity was 97% and 92.7%, respectively (42). Therefore, combined multitarget detection might be a promising method to improve sensitivity and specificity for cancer screening.

However, how to choose a candidate gene and how to interpret the findings remains to be clarified. In our study, we used a two-step screening strategy to select methylation biomarkers. The reason for choosing LASSO regression for feature selection is that LASSO is a linear model that estimates sparse coefficients. Especially in clinical diagnostic research, LASSO is widely used to drop disrelated variates to reduce the number of features and recover the exact set of non-zero coefficients *via* regularization. Subsequently, the prediction model for early screening of CRC was constructed using SVM, which can solve practical problems including small samples, nonlinearity, high dimensionality, and subdivided the methylation result into two categories (negative/positive methylation status) to determine the best separation threshold (43). Its core procedure was that the learning machine could adapt to the limited training of samples to obtain a robust prediction model. This robustness allows the addition and deletion of non-supported vector samples that have no effect on the model. During the construction of the model, we converted the Ct value to ‘0/1’ values according to the corresponding threshold and compared the impact of the converted and non-converted data on the accuracy of the prediction model. The sensitivity and specificity reached 97.80% and 94.80%, respectively using the Ct values; however, the prediction result exhibited by slight variations, which may have been caused by different settings of the threshold or baseline values.

To our knowledge, this is the first report on the use of methylated SDC2, TFPI2, WIF1, and NDRG4 genes for early detection of GCC, which not only retains the advantages of simple and non-invasive characteristics similar to FOBT, but also higher sensitivity and specificity to the screening or auxiliary diagnosis of a high-risk population with GCC. At the same time, due to the convenient sampling (home collection) and cheap costs ($100-300/test), it can be tested repeatedly in clinical application, which is conducive to continuous monitoring. In the future, a screening method with stool examination as the primary screening and endoscopy as confirmation will be conducive to popularization and optimization of GCC screening to further improve the detection rate of early GC and CRC. In addition to being able to directly detect GCC, the ColoCaller test can further stratify a high-risk population to improve endoscopy compliance, and promote timely diagnosis and treatment. However, at present, the sample size used in this study to verify the performance of the ColoCaller test was limited, and additional research data is still needed, especially due to the lack of patients with AA, furthermore, long-term follow-up data is needed to support our findings. Additionally, the detailed clinicopathological characteristics of patients with GCC was not included in this study, whether different disease sites or different molecular subtypes which may underestimate the diagnostic performance.

## Data Availability Statement

The datasets presented in this study can be found in online repositories. The names of the repository/repositories and accession number(s) can be found in the article/**Supplementary Material**.

## Ethics Statement

This study was approved by the Institutional Review Board of the China-Japan Friendship Hospital (No. No. 2019-50-Q07), China, and all patients provided written informed consent. The patients/participants provided their written informed consent to participate in this study.

## Author Contributions

Study design: LM, SS, and YC. Patient enrolment and patient data collection: SS, MZ, XK, PG, YJ, and YL. Experiment perform and Data analysis: JG, XF, LM, MZ, and XK. Manuscript preparation: JG, SS, YC, and LM. All authors discussed the results and implications, and critically revised and approved the final manuscript version.

## Funding

This study was funded by the Elite Medical Professionals project of China-Japan Friendship Hospital (Grant No : ZRJY2021-GG03); Key Clinical Specialty Project of Beijing (Grant No: 2020); National Natural Science Foundation of China (Grant No: 82074221).

## Conflict of Interest

JG and XF were employed by Apexbio Biotechnology (Suzhou) Co., Ltd.

The remaining authors declare that the research was conducted in the absence of any commercial or financial relationships that could be construed as a potential conflict of interest.

## Publisher’s Note

All claims expressed in this article are solely those of the authors and do not necessarily represent those of their affiliated organizations, or those of the publisher, the editors and the reviewers. Any product that may be evaluated in this article, or claim that may be made by its manufacturer, is not guaranteed or endorsed by the publisher.

## References

[1] Siegel RL Miller KD Fuchs HE Jemal A. Cancer Statistics, 2021. CA Cancer J Clin (2021) 71(1):7–33. doi: 10.3322/caac.21654 33433946

[2] Cao W Chen HD Yu YW Li N Chen WQ. Changing Profiles of Cancer Burden Worldwide and in China: A Secondary Analysis of the Global Cancer Statistics 2020. Chin Med J (Engl) (2021) 134(7):783–91. doi: 10.1097/CM9.0000000000001474 PMC8104205 33734139

[3] Rawla P Sunkara T Barsouk A. Epidemiology of Colorectal Cancer: Incidence, Mortality, Survival, and Risk Factors. Przeglad Gastroenterologiczny (2019) 14(2):89–103. doi: 10.5114/pg.2018.81072 31616522 PMC6791134

[4] Shi JF Wang L Ran JC Wang H Liu CC Zhang HZ. Clinical Characteristics, Medical Service Utilization, and Expenditure for Colorectal Cancer in China, 2005 to 2014: Overall Design and Results From a Multicenter Retrospective Epidemiologic Survey. Cancer (2021) 127(11):1880–93. doi: 10.1002/cncr.33445 33784413

[5] Gurzu S Jung I Kadar Z. Aberrant Metastatic Behavior and Particular Features of Early Gastric Cancer. APMIS (2015) 123(12):999–1006. doi: 10.1111/apm.12469 26547366

[6] Armaghany T Wilson JD Chu Q Mills G. Genetic Alterations in Colorectal Cancer. Gastrointest Cancer Res (2012) 5(1):19–27. 22574233 PMC3348713

[7] Jones PA. Functions of DNA Methylation: Islands, Start Sites, Gene Bodies and Beyond. Nat Rev Genet (2012) 13(7):484–92. doi: 10.1038/nrg3230 22641018

[8] de Souza CF Sabedot TS Malta TM Stetson L Morozova O Sokolov A. A Distinct DNA Methylation Shift in a Subset of Glioma CpG Island Methylator Phenotypes During Tumor Recurrence. Cell Rep (2018) 23(2):637–51. doi: 10.1016/j.celrep.2018.03.107 PMC8859991 29642018

[9] Sobanski T Arantes L Dos Santos W Matsushita M de Oliveira MA Costa M. Methylation Profile of Colon Cancer Genes in Colorectal Precursor Lesions and Tumor Tissue: Perspectives for Screening. Scand J Gastroenterol (2021) 56(8):920–8. doi: 10.1080/00365521.2021.1922744 34218733

[10] Xu M Yuan L Wang Y Chen S Zhang L Zhang X. Integrative Analysis of DNA Methylation and Gene Expression Profiles Identifies Colorectal Cancer-Related Diagnostic Biomarkers. Pathol Oncol Res (2021) 27:1609784. doi: 10.3389/pore.2021.1609784 34366718 PMC8333028

[11] Iyengar V Albaugh GP Lohani A Nair PP. Human Stools as a Source of Viable Colonic Epithelial Cells. FASEB J (1991) 5(13):2856–9. doi: 10.1096/fasebj.5.13.1655550 1655550

[12] Ratto C Flamini G Sofo L Nucera P Ippoliti M Curigliano G. Detection of Oncogene Mutation From Neoplastic Colonic Cells Exfoliated in Feces. Dis Colon Rectum (1996) 39(11):1238–44. doi: 10.1007/BF02055116 8918432

[13] Mojtabanezhad Shariatpanahi A Yassi M Nouraie M Sahebkar A Varshoee Tabrizi F Kerachian MA. The Importance of Stool DNA Methylation in Colorectal Cancer Diagnosis: A Meta-Analysis. PloS One (2018) 13(7):e0200735. doi: 10.1371/journal.pone.0200735 30024936 PMC6053185

[14] Ahlquist T Lind GE Costa VL Meling GI Vatn M Hoff GS. Gene Methylation Profiles of Normal Mucosa, and Benign and Malignant Colorectal Tumors Identify Early Onset Markers. Mol Cancer (2008) 7:94. doi: 10.1186/1476-4598-7-94 19117505 PMC2639620

[15] Muller HM Oberwalder M Fiegl H Morandell M Goebel G Zitt M. Methylation Changes in Faecal DNA: A Marker for Colorectal Cancer Screening? Lancet (2004) 363(9417):1283–5. doi: 10.1016/S0140-6736(04)16002-9 15094274

[16] Ahlquist DA Zou H Domanico M Mahoney DW Yab TC Taylor WR. Next-Generation Stool DNA Test Accurately Detects Colorectal Cancer and Large Adenomas. Gastroenterology (2012) 142(2):248–56; quiz e25-6. doi: 10.1053/j.gastro.2011.10.031 22062357 PMC4017869

[17] Imperiale TF Ransohoff DF Itzkowitz SH. Multitarget Stool DNA Testing for Colorectal-Cancer Screening. N Engl J Med (2014) 371(2):187–8. doi: 10.1056/NEJMc1405215 25006736

[18] Wang J Liu S Wang H Zheng L Zhou C Li G. Robust Performance of a Novel Stool DNA Test of Methylated SDC2 for Colorectal Cancer Detection: A Multicenter Clinical Study. Clin Epigenet (2020) 12(1):162. doi: 10.1186/s13148-020-00954-x PMC7602331 33126908

[19] Song L Jia J Peng X Xiao W Li Y. The Performance of the SEPT9 Gene Methylation Assay and a Comparison With Other CRC Screening Tests: A Meta-Analysis. Sci Rep (2017) 7(1):3032. doi: 10.1038/s41598-017-03321-8 28596563 PMC5465203

[20] Force USPST Davidson KW Barry MJ Mangione CM Cabana M Caughey AB. Screening for Colorectal Cancer: US Preventive Services Task Force Recommendation Statement. JAMA (2021) 325(19):1965–77. doi: 10.1001/jama.2021.6238 34003218

[21] Cao Y Zhao G Yuan M Liu X Ma Y Cao Y. KCNQ5 and C9orf50 Methylation in Stool DNA for Early Detection of Colorectal Cancer. Front Oncol (2020) 10:621295. doi: 10.3389/fonc.2020.621295 33585248 PMC7878552

[22] Zhang W Yang C Wang S Xiang Z Dou R Lin Z. SDC2 and TFPI2 Methylation in Stool Samples as an Integrated Biomarker for Early Detection of Colorectal Cancer. Cancer Manag Res (2021) 13:3601–17. doi: 10.2147/CMAR.S300861 PMC8096344 33958894

[23] Zhang X Wan S Yu Y Ruan W Wang H Xu L. Identifying Potential DNA Methylation Markers in Early-Stage Colorectal Cancer. Genomics (2020) 112(5):3365–73. doi: 10.1016/j.ygeno.2020.06.007 32531444

[24] Li LC Dahiya R. MethPrimer: Designing Primers for Methylation PCRs. Bioinformatics (2002) 18(11):1427–31. doi: 10.1093/bioinformatics/18.11.1427 12424112

[25] Liu H Gao L Xie T Li J Zhai TS Xu Y. Identification and Validation of a Prognostic Signature for Prostate Cancer Based on Ferroptosis-Related Genes. Front Oncol (2021) 11:623313. doi: 10.3389/fonc.2021.623313 34336641 PMC8320699

[26] Das P Roychowdhury A Das S Roychoudhury S Tripathy S. Sigfeature: Novel Significant Feature Selection Method for Classification of Gene Expression Data Using Support Vector Machine and T Statistic. Front Genet (2020) 11:247. doi: 10.3389/fgene.2020.00247 32346383 PMC7169426

[27] Meyer DDE Hornik K Weingessel A Leisch F. E1071: Misc Functions of the Department of Statistics, Probability Theory Group (Formerly: E1071), TU Wien. R Packag. Version 1. (2017) Available at: https://CRAN.Rproject.org/package=e1071.

[28] Puig I López-Cerón M Arnau A Rosiñol Ò Cuatrecasas M Herreros-de-Tejada A. Accuracy of the Narrow-Band Imaging International Colorectal Endoscopic Classification System in Identification of Deep Invasion in Colorectal Polyps. Gastroenterology (2019) 156(1):75–87. doi: 10.1053/j.gastro.2018.10.004 30296432

[29] Chen H Li N Ren J Feng X Lyu Z Wei L. Participation and Yield of a Population-Based Colorectal Cancer Screening Programme in China. Gut (2019) 68(8):1450–7. doi: 10.1136/gutjnl-2018-317124 30377193

[30] National Cancer Center, China Expert Group of the Development of China Guideline for the Screening. Early Detection and Early Treatment of Colorectal Cancer. China Guideline for the Screening, Early Detection and Early Treatment of Colorectal Cancer (2020, Beijing). Zhonghua Zhong Liu Za Zhi (2021) 43(1):16–38. doi: 10.3760/cma.j.cn112152-20210105-00010 33472315

[31] National Clinical Research Center for Digestive D National Early Cancer Prevention and Treatment Centers of Digestive Tract Digestive Endoscopy Branch of Chinese Medical Association Chinese Medical Association Health Management Association Digestive Endoscopy Committee of Endoscopy Branch of Chinese Medical Doctor Association, Endoscopy Health Management and Physical Examination Committee of Endoscopy Branch of Chinese Medical Doctor Association. Chinese Consensus of Early Colorectal Cancer Screening (2019, Shanghai). Zhonghua Nei Ke Za Zhi (2019) 58(10):736–44. doi: 10.3760/cma.j.issn.0578-1426.2019.10.004

[32] Lou S Shaukat A. Noninvasive Strategies for Colorectal Cancer Screening: Opportunities and Limitations. Curr Opin Gastroenterol (2021) 37(1):44–51. doi: 10.1097/MOG.0000000000000688 33074994

[33] Mo S Wang H Han L Xiang W Dai W Zhao P. Fecal Multidimensional Assay for Non-Invasive Detection of Colorectal Cancer: Fecal Immunochemical Test, Stool DNA Mutation, Methylation, and Intestinal Bacteria Analysis. Front Oncol (2021) 11:643136. doi: 10.3389/fonc.2021.643136 33718241 PMC7947614

[34] Watanabe Y Kim HS Castoro RJ Chung W Estecio MR Kondo K. Sensitive and Specific Detection of Early Gastric Cancer With DNA Methylation Analysis of Gastric Washes. Gastroenterology (2009) 136(7):2149–58. doi: 10.1053/j.gastro.2009.02.085 PMC2722957 19375421

[35] Goel A. DNA Methylation-Based Fecal Biomarkers for the Noninvasive Screening of GI Cancers. Future Oncol (2010) 6(3):333–6. doi: 10.2217/fon.10.9 20222788

[36] Hu H Chen X Wang C Jiang Y Li J Ying X. The Role of TFPI2 Hypermethylation in the Detection of Gastric and Colorectal Cancer. Oncotarget (2017) 8(48):84054–65. doi: 10.18632/oncotarget.21097 PMC5663576 29137404

[37] Hibi K Goto T Shirahata A Saito M Kigawa G Nemoto H. Detection of TFPI2 Methylation in the Serum of Gastric Cancer Patients. Anticancer Res (2011) 31(11):3835–8. doi: 10.1016/j.canlet.2011.07.006 22110206

[38] Guo M Li W Li B Zou B Wang S Fan B. Multiple Immune Features-Based Signature for Predicting Recurrence and Survival of Inoperable LA-NSCLC Patients. Front Oncol (2020) 10:571380. doi: 10.3389/fonc.2020.571380 33154945 PMC7591766

[39] Lee SW Lee HY Bang HJ Song HJ Kong SW Kim YM. An Improved Prediction Model for Ovarian Cancer Using Urinary Biomarkers and a Novel Validation Strategy. Int J Mol Sci (2019) 20(19):4938. doi: 10.3390/ijms20194938 PMC6801627 31590408

[40] Yin XH Yu LP Zhao XH Li QM Liu XP He L. Development and Validation of a 4-Gene Combination for the Prognostication in Lung Adenocarcinoma Patients. J Cancer (2020) 11(7):1940–8. doi: 10.7150/jca.37003 PMC7052877 32194805

[41] Zhang JX Song W Chen ZH Wei JH Liao YJ Lei J. Prognostic and Predictive Value of a microRNA Signature in Stage II Colon Cancer: A microRNA Expression Analysis. Lancet Oncol (2013) 14(13):1295–306. doi: 10.1016/S1470-2045(13)70491-1 24239208

[42] Zhai RL Xu F Zhang P Zhang WL Wang H Wang JL. The Diagnostic Performance of Stool DNA Testing for Colorectal Cancer: A Systematic Review and Meta-Analysis. Med (Baltimore) (2016) 95(5):e2129. doi: 10.1097/MD.0000000000002129 PMC4748866 26844449

[43] Mortazavi BJ Bucholz EM Desai NR Huang C Curtis JP Masoudi FA. Comparison of Machine Learning Methods With National Cardiovascular Data Registry Models for Prediction of Risk of Bleeding After Percutaneous Coronary Intervention. JAMA Netw Open (2019) 2(7):e196835. doi: 10.1001/jamanetworkopen.2019.6835 31290991 PMC6624806

