# Supplementary figures and images for: A Novel Stool Methylation Test for the Non-Invasive Screening of Gastric and Colorectal Cancer
Source: Front Oncol. 2022 Mar 28;12:860701. doi: 10.3389/fonc.2022.860701 (PMC8995552; doi:10.3389/fonc.2022.860701)

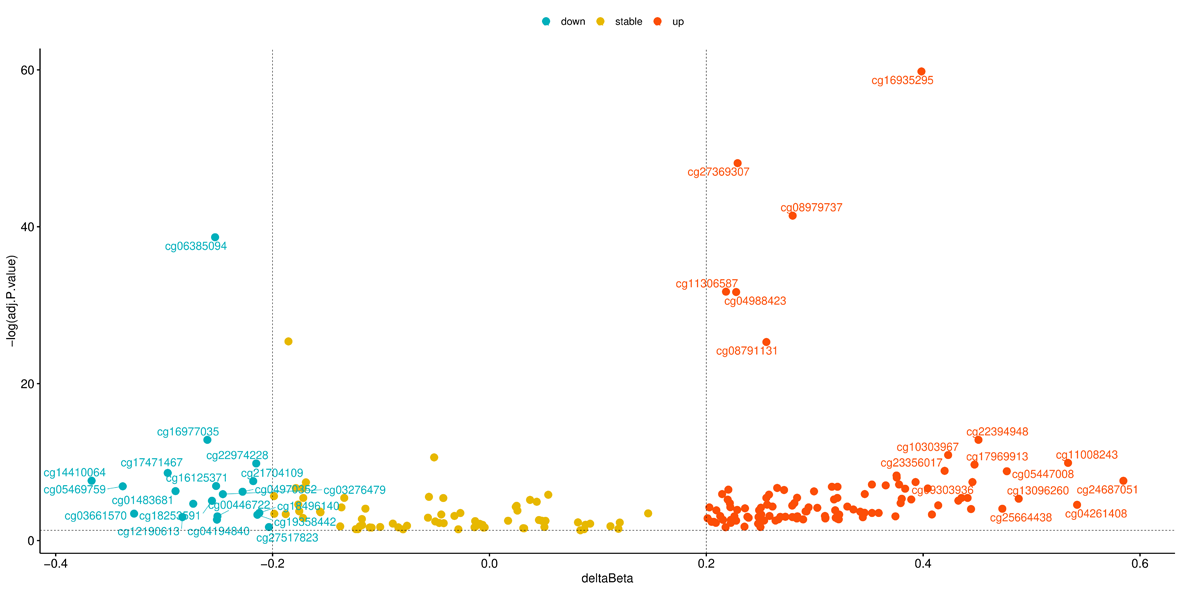

Supplement: Supplementary file 1 [file Image_1.tif]

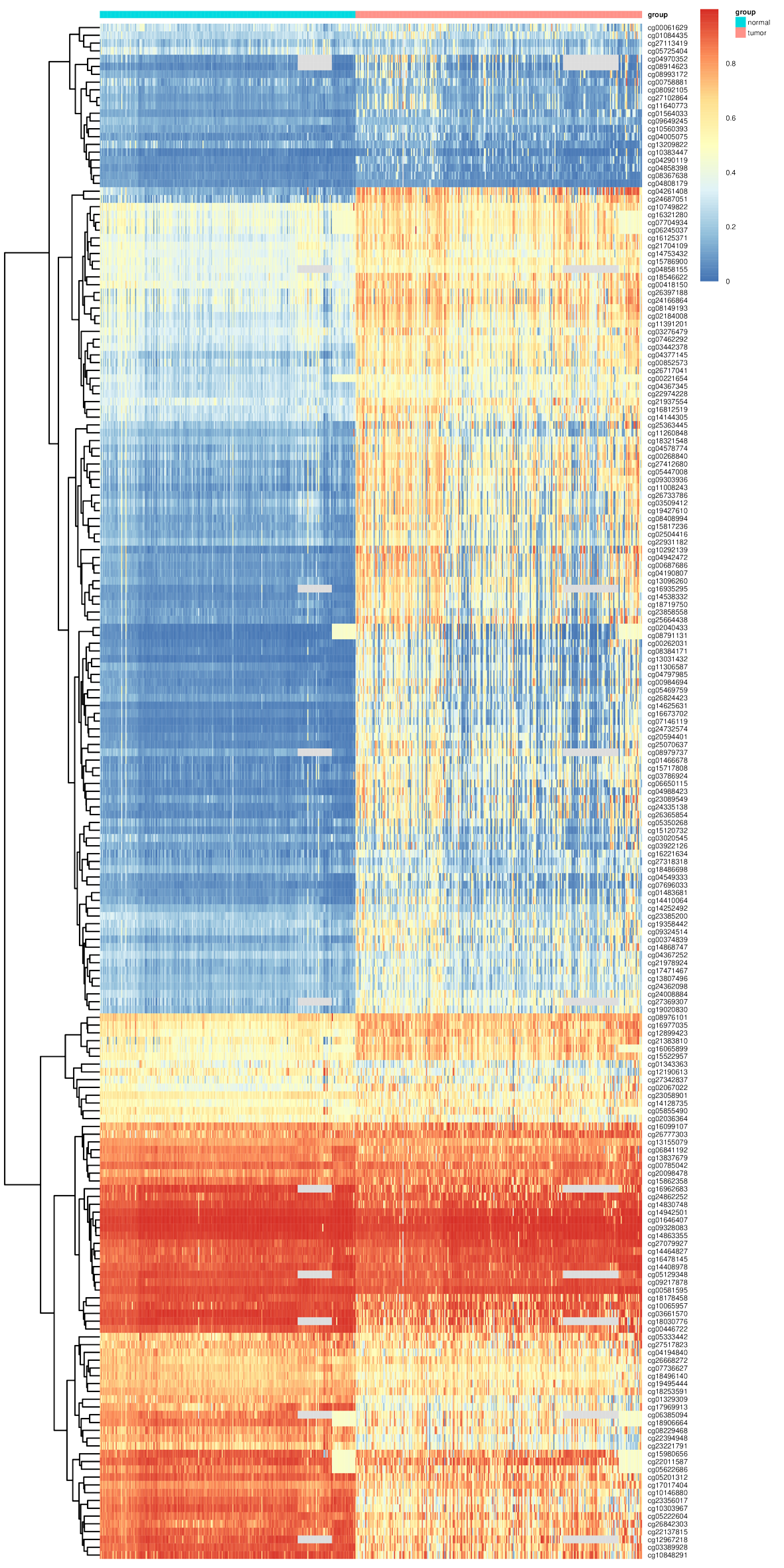

Supplement: Supplementary file 2 [file Image_2.tif]

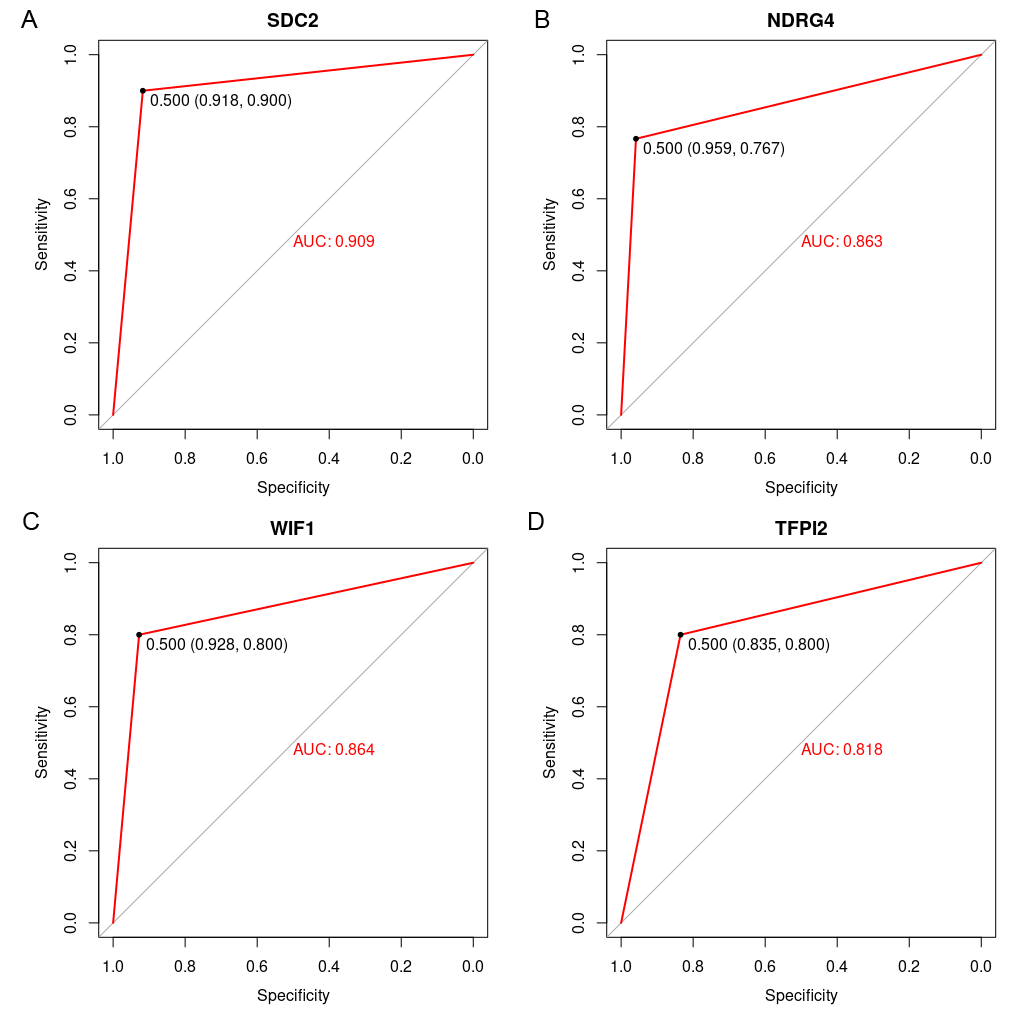

Supplement: Supplementary file 3 [file Image_3.tif]

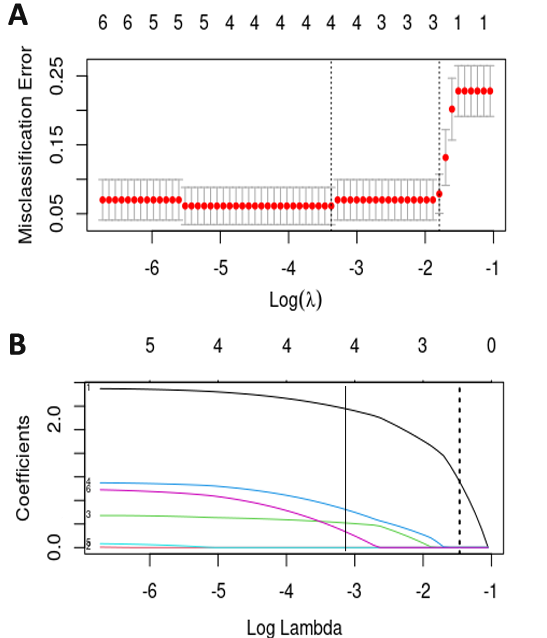

Supplement: Supplementary file 4 [file Image_4.tif]
